# Computational Analysis of Dengue Virus Envelope Protein (E) Reveals an Epitope with Flavivirus Immunodiagnostic Potential in Peptide Microarrays

**DOI:** 10.3390/ijms20081921

**Published:** 2019-04-18

**Authors:** Greta Bergamaschi, Enrico M. A. Fassi, Alessandro Romanato, Ilda D’Annessa, Maria Teresa Odinolfi, Dario Brambilla, Francesco Damin, Marcella Chiari, Alessandro Gori, Giorgio Colombo, Marina Cretich

**Affiliations:** 1 Consiglio Nazionale delle Ricerche, Istituto di Chimica del Riconoscimento Molecolare (ICRM), Via Mario Bianco 9, 20131 Milano, Italy; gretabergamaschi@gmail.com (G.B.); fassi.enrico@yahoo.it (E.M.A.F.); alessandro.romanato@icrm.cnr.it (A.R.); dnnldi00@uniroma2.it (I.D.); tea.odinolfi@gmail.com (M.T.O.); dario.brambilla@icrm.cnr.it (D.B.); francesco.damin@icrm.cnr.it (F.D.); marcella.chiari@icrm.cnr.it (MA.C.); alessandro.gori@icrm.cnr.it (A.G.); 2 Dipartimento di Chimica, Università di Pavia, Via Taramelli 12, 27100 Pavia, Italy

**Keywords:** Dengue virus, epitope, antibody, infectious diseases, microarrays, diagnostics

## Abstract

The mosquito-borne viral disease caused by the Dengue virus is an expanding global threat. Diagnosis in low-resource-settings and epidemiological surveillance urgently requires new immunoprobes for serological tests. Structure-based epitope prediction is an efficient method to design diagnostic peptidic probes able to reveal specific antibodies elicited in response to infections in patients’ sera. In this study, we focused on the Dengue viral envelope protein (E); computational analyses ranging from extensive Molecular Dynamics (MD) simulations and energy-decomposition-based prediction of potentially immunoreactive regions identified putative epitope sequences. Interestingly, one such epitope showed internal dynamic and energetic properties markedly different from those of other predicted sequences. The epitope was thus synthesized as a linear peptide, modified for chemoselective immobilization on microarrays and used in a serological assay to discriminate Dengue-infected individuals from healthy controls. The synthetic epitope probe showed a diagnostic performance comparable to that of the full antigen in terms of specificity and sensitivity. Given the high level of sequence identity among different flaviviruses, the epitope was immune-reactive towards Zika-infected sera as well. The results are discussed in the context of the quest for new possible structure-dynamics-based rules for the prediction of the immunoreactivity of selected antigenic regions with potential pan-flavivirus immunodiagnostic capacity.

## 1. Introduction

Dengue fever is the most important arthropod-borne viral infection in humans; its transmission occurs mostly in urban and semi-urban areas of tropical and sub-tropical climates and has expanded worldwide in recent years [1]. Dengue causes flu-like illness but occasionally develops into severe dengue fever, a potentially lethal complication also known as dengue hemorrhagic fever that has become a leading cause of hospitalization and death among children and adults in endemic areas [2]. There is no specific treatment for dengue fever and for severe dengue disease, but early detection and access to proper medical care lowers fatality rates below 1% [3].

Serological assays are commonly used for diagnosis of dengue infection as these assays are relatively inexpensive and easy to perform compared with culture or nucleic acid-based methods. Even though it is not relevant for diagnosis of the early stages, the detection of dengue-specific immune-response to past infections is of the utmost importance for epidemiological surveillance and to evaluate the risk of severe dengue. This risk is related to the circulation of 4 distinct serotypes of dengue virus: DEN-1, DEN-2, DEN-3 and DEN-4. Recovery from one infection provides lifelong immunity against that particular serotype. However, subsequent infections by other serotypes increase the risk of developing severe dengue because cross-immunity to the other serotypes is only partial and temporary [4,5]. Risk of severe dengue should be evaluated also in the context of vaccination campaigns. For example, the World Health Organization (WHO) recommends pre-vaccination screenings to immunize only individuals with evidence of a past dengue infection (seropositive) due to the increased risk of severe dengue in seronegative individuals [3,5].

In this context, the availability of cheap, reliable and simple immune-assays to spread the use of serological tests for dengue even in low-resource settings is a pressing necessity also in light of the fact that the actual numbers of dengue cases are still underreported or misclassified [3].

Current tests to detect dengue nonstructural protein 1 (NS1) antigen and patient’s antibodies are widely used and provide acceptable levels of detection sensitivity and specificity [6,7]. However, these assays are instrument-dependent and require laboratory facilities. In contrast, rapid diagnostic tests (RDTs) based on lateral and vertical flow and paper-based dipstick assays are ideal tools for de-centralized diagnostics, wherever fully-equipped facilities and highly trained medical staff are lacking. The performance of the available tests needs, nonetheless, to be improved in order to increase detection sensitivity and to achieve equal diagnostic performance across all dengue serotypes [8]. RDTs for patient’s antibody detection are based mostly on the use of poorly characterized crude viral extracts or on recombinant NS1 full antigen, making both approaches liable to probe instability and irreproducibility issues [7,9,10].

Synthetic peptides mimicking the antigenic determinants of full protein antigens and acting as their minimized surrogates, offer new opportunities for the development of immunoprobes with superior characteristics in terms of ease of handling, reproducibility, costs and stability in the typical low resource setting conditions (for example storage at high humidity at RT) [11,12,13].

In this framework, we aimed at the development of new and more efficient dengue peptidic immunoprobes that can be aptly implemented in RDTs. We focused on the dengue envelope (E) protein, since the important biological properties of dengue viruses, including the induction of neutralizing antibodies and protective immune responses, are associated with this glycoprotein [1].

The envelope protein (E), which forms the outermost layer of the virion particle, is one of the key players in the phenomenon of viral breathing, i.e., the reversible transition among conformations with different degrees of compactness, caused by temperature-increases and it responds with conformational changes to pH variations occurring during infection as the virus is transported into endosomes.

We performed a computational analysis on protein (E) that combines energetic and conformational dynamic profiling, illuminating potentially immunoreactive regions. We predicted putative epitopes that were synthesized as linear peptides and used as capturing probes in serological tests on dengue-affected individuals. Their immunodiagnostic performance is compared with that of the full protein E. Finally, we propose our approach as a viable route to correlate the dynamics and energetic properties of antigenic proteins to their observed diagnostic performances.

## 2. Results and Discussion

### 2.1. Computational Analyses

**Figure 1:**
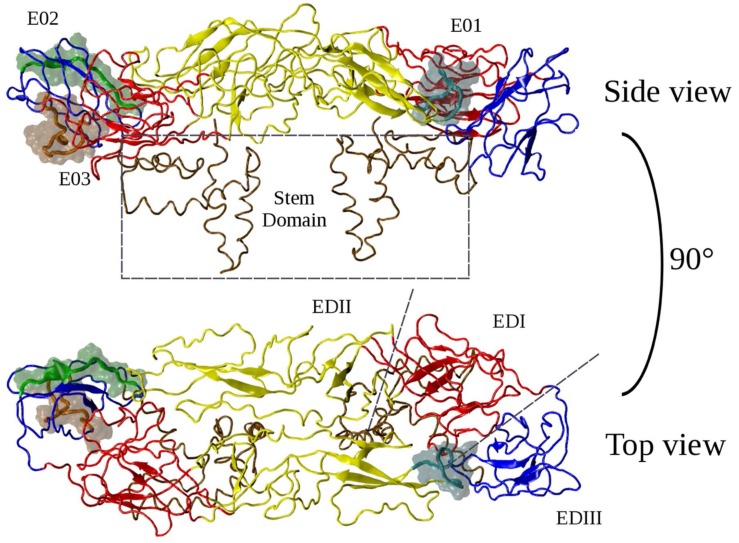
Structure of the envelope (E) protein and localization of the putative epitopes E01, E02, E03.

From a structural point of view, the E protein is composed of four domains: E domain (ED)I (residues 1–52, 132–193, 280–296), EDII (residues 53–131, 194–279), EDIII (residues 297–394), which form the globular shape of the protein that lies on the lipidic surface, while a stem domain (residues 395–486) connects the EDI–EDIII domains to the transmembrane region [14] (Figure 1). 

The EDI domain is characterized by an eight-stranded β-barrel structure and acts as a bridge connecting EDII with EDIII [15]. EDI harbors the N-linked glycosylation site Asn153, conserved in most flaviviruses [14]. EDII is responsible for the stabilization of the dimer and contains a second glycosylation site, Asn67, and more importantly the fusion loop, a stretch spanning amino acids 98–110 responsible for the fusion between viral and host membranes during infection [16]. Finally, EDIII is characterized by an immunoglobulin-like structure formed by six anti-parallel β-strands and anchored to the C-terminal stem domain. Because of its high flexibility the domain is relatively independent from the rest of the protein and is thus considered to interact with host membrane receptors to start the infection process.

To predict candidate epitope sequences for subsequent testing, we ran the Matrix of Local Coupling Energies method (MLCE) method on the crystal structure of the protein and on different representatives of the most populated clusters of conformations visited during Molecular Dynamics (MD) simulations (namely, clusters 1 and 2, representative of more than 90% of the total conformational ensemble). MLCE integrates the analysis of the dynamical and energetic properties of proteins to identify non-optimized, low-intensity energetic interaction-networks on the surface of the isolated antigen. Such regions have been proven in several cases to correspond to substructures that can be recognized by an antibody. The main idea of the method is that the structure, dynamics and stability of a protein-antigen play a key role in the interaction with antibodies.

**Figure 2:**
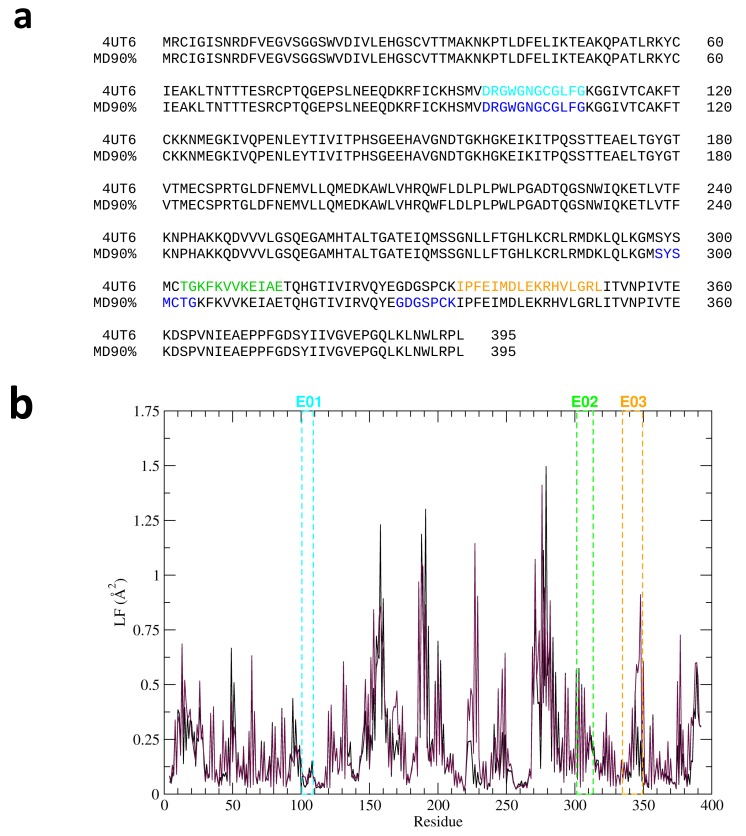
(**a**) The sequences of predicted epitopes are shown in color on the sequence of the full-length protein. The predictions on the crystal structure (line 4UT6) and on the cluster representative recapitulating 90% of the visited structures are shown. It is interesting to see that only E01, sequence DRGWGNGCGLFG, is predicted with no variation as an epitope in the two cases. (**b**) Local flexibility of each residue in each of the two monomers of the E protein. The predicted epitope regions are indicated with colored broken lines.

In the various structures considered herein (namely the crystal and different representatives from MD trajectories), predictions were carried out on the whole protein as well as on the separated dimers, considered as isolated entities in the solution. The comparative analysis of the results showed that only epitope E01 (see Table 1) could be predicted as an epitope in all the structures considered, from the crystal structure to the cluster representatives (See Figure 2a) Other putative epitopes predicted from the crystal structures, namely E02 and E03, could not be confirmed when considering the full dynamics of the protein.

To gain more insights into the molecular reasons why E01 could be consistently predicted as an epitope in different structural representatives of the protein, while E02 and E03 could not, we set out to characterize the conformational dynamic properties of the putative epitopes. We characterized residue-flexibility modulation throughout the whole protein by means of local fluctuation (LF) analysis (Figure 2b), to focus on changes at the residue-level resolution as they may report on how structural changes in specific regions may underpin antibody-recognition.

LF informs on the fluctuations of the distances between any pair of residues in the ensemble (*i, i* ± 2), where *i* is the sequence number, highlighting structural deformations that involve contiguous sequence stretches. LF thus identifies protein areas undergoing significant microscopic rearrangements. Namely, regions involved in conformational changes show LF peaks, which can be related to the degree of local deformation experienced by the protein during its normal breathing in solution. Interestingly, E01 showed a significantly different behavior from E02 and E03: while LF in E01 tend to be low, E02 and E03 undergo sizeable local distortions. In this framework, it is tempting to suggest that E01 is energetically decoupled from the rest of the protein while showing a significant structural preorganization potentially favorable for antibody recognition, as indicated by the low degree of local flexibility.

To investigate whether different structure-dynamics-related properties among the three putative epitopes could reverberate in distinct immunoreactivity behavior, we synthesized their respective sequences and tested their behavior in a microarray setting.

### 2.2. Immunoreactivity Tests

Microarray immunoreactivity tests to verify the diagnostic potential of the predicted epitopes were developed as previously reported [17,18,19] by the chemo-selective immobilization of *yne*-modified peptides on copoly Azide coated silicon slides [17] via the azide-alkyne-cycloaddition (CuACC). An array of E01, E02 and E03 peptides was tested with a panel of sera from 20 Dengue affected individuals and 20 healthy controls. Samples were incubated on the peptide array to detect infection-specific IgG as described in the experimental section. The ability of each epitope to distinguish between controls and patients was tested on microarrays by the detection of the peptide-specific fluorescence signals provided by healthy individuals and dengue patients; the significance of the results was statistically evaluated performing the unpaired t test. Accuracy in discriminating infected individuals vs. healthy controls was evaluated quantitatively by a Receiver Operating Characteristic (ROC) curve analysis and *t* test analysis.

**Figure 3:**
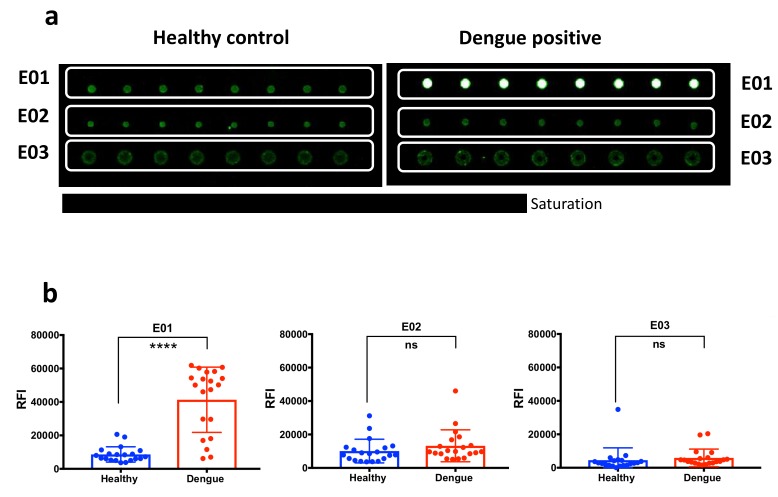
(**a**) Spotting scheme and fluorescence images for the analysis of a representative dengue positive serum and a healthy control. Image acquired at 50% laser power and 10% Photo-Multiplier (PMT) gain. (**b**) Scatter plots reporting individual and mean immunoreactivity with standard deviation (SD) of dengue positive and healthy control individuals and results of the unpaired *t* Test dengue infection diagnosis. ns = not significant. Significant: **** *p* < 0.0001.

**Figure 4:**
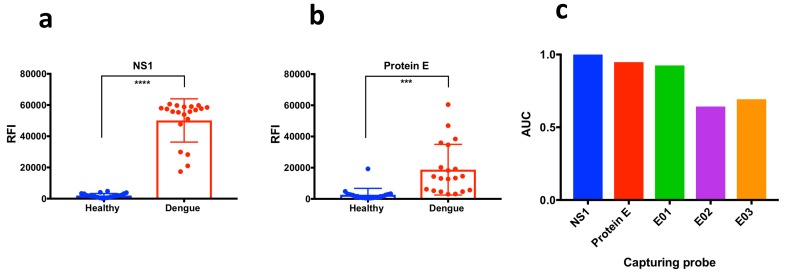
Scatter plots reporting individual and mean immunoreactivity with SD of dengue positive and healthy control individuals and results of the unpaired *t* Test dengue infection diagnosis for NS1 (**a**) and protein E (**b**). ns = not significant. Significant: *** *p* < 0.001; **** *p* < 0.0001. (**c**) Comparison of Area under the Receiver Operating Characteristic (ROC) curve (area under the curve, AUC) for the different capturing probes. nonstructural protein 1 (NS1):1; Protein E: 0.9475; E01: 0.925; E02: 0.5225; E03: 0.61. For NS1 antigen the sensitivity and specificity of the test was 100% (95% CI: 83,16% to 100%) and 75% (95% CI: 50.9% to 91.34%) respectively. For the E protein the sensitivity and specificity of the test was 95% (95% CI: 75,13% to 99.87%) and 75% (95% CI: 50.9% to 91.34%) respectively. For E01 the sensitivity and specificity of the test was 100% (95% CI: 83.168% to 100%) and 75% (95% CI: 50.9% to 91.34%) respectively. ROC curves for NS1, protein E and E01 are reported in Figure S2 in the Supplementary Material. CI: Confidence Interval.

Figure 3a reports the spotting scheme and representative fluorescence images of the microarrays after incubation with a dengue sample and a healthy control. In general, peptide E01 showed a higher immunoreactivity in comparison to E02 and E03 and resulted effective in distinguishing dengue positive patients from negative controls, as shown in Figure 3b by the statistical evaluation of the diagnostic performance of the three arrayed peptides. The peptide E01 discriminates the two patient’s groups with *p* < 0.0001 determined by unpaired t test. Accordingly, the evaluation of its diagnostic specificity and sensitivity provided an area under the curve (AUC) equal to 0.925. In accordance with the computational studies, E02 and E03 were non-effective in discriminating the two sample panels and provided AUC equal to 0.5225 and 0.61 respectively (Figure 4c).

Given the high level of sequence conservation and identity among different flavivirus, including Zika virus, we hypothesized that peptide E01 could have immune-diagnostic properties also for other flavivirus infections. The three peptides were, therefore, tested with 16 Zika positive samples. As expected, E01 was highly reactive, providing a diagnostic capability with *p* < 0.0001, the same as that obtained with Dengue positive individuals (Scatter plots reported in Figure S1).

The analytical performance of the isolated epitope E01 was then compared to that provided by the full protein E; NS1 antigen was also tested as a diagnostic benchmark. A protein microarray displaying the two recombinant antigens was developed as reported in the experimental section; the diagnostic performance of the proteins in discriminating Dengue positive patients from negative controls is reported in Figure 4. The proteins were effective in distinguishing the two patient’s groups with *p* < 0.0001 for NS1 and *p* < 0.001 for protein E as determined by unpaired *t* test (Figure 4a). By analysing the ROC curves, the protein antigens provided an area under the curve (AUC) equal to 1 and 0.945 for NS1 and protein E respectively (Figure 4c)

Overall, the diagnostic performances of the two recombinant antigens were comparable to that provided by isolated epitope E01. In particular, NS1 antigen performed as the most specific and sensitive capturing probe (AUC equal to 1). This result is in line with the widespread use of the NS1 antigen in many immunodiagnostic assays such as ELISA and immunochromatographic tests. The full antigen E and its isolated epitope E01 provided very similar AUC values (0.9475 and 0.925 respectively). Notably, the statistical significance of the unpaired t test resulted to be higher for peptide E01 (*p* < 0.0001) than for protein E (*p* < 0.001). Even if obtained using a limited panel of samples, these results confirm the feasibility of the use in serological tests of properly designed immunoreactive peptides as substitutes of full antigens with comparable sensitivity and specificity.

Antigenic peptides indeed represent a valuable alternative to recombinant proteins (and to crude pathogen extracts) for their improved characteristics in terms of batch-to-batch production reproducibility, ease of purification and handling, cost and stability. Peptides offer the advantage of eliminating the possible cross-reactivity with antigens copurified with recombinant products. However, sometimes synthetic peptides lose their antigenicity, when immunoreactive residues are hidden by non-specifically adsorption to the analytical support; furthermore, the surface binding efficiency of a synthetic peptide is dependent on its size which is sometimes reflecting into difficulties of achieving a high degree of steric and chemical fit between the peptide antigen and the target antibody surfaces. In this framework, peptide synthetic accessibility combined with the large chemical diversity that can be implemented in such products is particularly relevant allowing to develop bio-conjugation methods for precise spatial arrangement of the aminoacidic sequence, optimal surface presentation onto diverse analytical platforms (microarrays, ELISA, RDTs, bead-based assays) and to gain enhanced immunoreactivity by multi-presentation strategies [19]. Another limitation to the use of peptides is due to the fact that the only epitopes that are readily mimicked by linear synthetic peptides are continuous epitopes corresponding to stretches of 6–10 residues in the protein antigen. The more common conformational epitopes, made of residues originating from different stretches on the antigen polypeptide chain, require antigen surface mimicry approaches based on macrocyclization [20,21,22,23] or spatially controlled co-presentation strategies [19]. In this direction, even more synthetically demanding efforts are needed when antigenic determinants rely on post-translational modifications, such as glycosylation, and when the formation of intramolecular covalent bonds or the use of anchoring macrocyclic rigid scaffolds to thermodynamically favor a limited set of peptide conformations are needed [24,25].

### 2.3. Conclusions

The efficient design and deployment of novel and easy-to-use diagnostic probes represents a relevant necessity for both practical and fundamental reasons. From a practical point of view, emergence and re-emergence of mosquito-borne arboviruses, such as the Dengue, Zika and Chikungunya viruses, are of great public health importance, resulting in numerous outbreaks worldwide. Efficient implementation of serodiagnosis in developing countries and small ambulatory care settings by low-cost and long shelf life diagnostic devices is mandatory to control the diseases. In this frame the use of peptide mimics able to recapitulate the diagnostic properties of the full-length antigen and to efficiently engage disease biomarkers is an attractive strategy towards the development of new materials for point-of-care (POC) tests. Furthermore, it is possible to envision that the combination of peptides with other biomolecules [26], as well as the integration with diverse presentation approaches based, for instance, on a multivalent display [27], may evolve into new and more efficient molecular diagnostic tools. From a fundamental point of view, understanding the molecular determinants of why certain regions on a protein antigen are immunoreactive and others are not, will further our understanding of the relationships between molecular recognition and functional responses in immunological processes.

Here we have shown the design, synthesis and application of a novel peptide-based molecule able to mimic the antigenic performance of the full dengue antigen E with the aim to demonstrate the potential of peptides as epitope-mimicry in the development of efficient diagnostic methods for infectious diseases. The predicted epitope E01 was not specific for Dengue fever: it showed similar immunodiagnostic performance in discriminating Zika infected individuals from healthy controls. As such, more tests would be needed to determine if a patient is affected by Dengue fever. Because the E01 sequence is identical in West Nile Fever Virus, Japanese Encephalitis Virus and Yellow Fever Virus, we expect this epitope to be a pan-flavivirus immunodiagnostic probe. Interestingly, the integration of energetic information with dynamic characterization, followed by experimental verification, suggests a possible rationale for E01 immunoreactivity. In this framework, E01 is energetically decoupled from the rest of the protein while undergoing low local structural deformations so that the epitope region appears to be preorganized for recognition by a second partner, the antibody. At the same time, the minimal coupling to the rest of the protein, revealed by MLCE, indicates that an antibody would not have to compete with strong intramolecular interactions to capture the antigen establishing productive interactions. It is worth noting here that E01 spans the “fusion loop”, used to establish contacts with the host membrane during infection. Furthermore, a recent investigation of the E protein by Sharma et al. [28] combining FRET, MS and MD analysis showed that the regions corresponding to E02 and E03 in our analyses, are involved in the binding and chelation of divalent metal ions, which may mask these epitope stretches from antibody recognition. From the point of view of the improvement of prediction methods, the recent emergence of quantum-mechanics-based approaches for the treatment of large biosystems holds great potential for optimizing epitope identification, keeping into account electronic factors that are somewhat neglected in classical mechanics approaches [29,30].

In conclusion, the differential immunoreactivities of the identified regions, together with their observed functional roles, support the validity of our integrated computational-experimental discovery method. It is important to underline here that, while based on the specific case of the E protein, the method we have described is fully general and immediately applicable to other cases. 

## 3. Materials and Methods

### 3.1. Samples

Serum samples were purchased from AbBaltisLtd (Sittingbourne, UK). Samples were characterized for the presence of Dengue positive IgM/IgG by tests performed by the purchaser. The Zika positive samples were from Boca Biolistics (Pompano Beach, FL, USA). Positivity for Zika infection was confirmed by MAC ELISA performed by the vendor.

### 3.2. Reagents and Procedures

Reagents for peptide synthesis were from Iris Biotech (Marktredwitz, Germany). Other chemicals were from Sigma-Aldrich (St. Louis, MO, USA) if not stated otherwise. Goat anti-human IgG labeled by Cy3 was obtained from Jackson Immunoresearch (West Grove, PA, USA).

Copoly Azide was obtained by post-polymerization modification reaction of copoly(DMA-NAS-MAPS) with 3-azido-1-propanamine as previously reported [18,19].

Silicon slides (SVM Sunnyvail, CA, USA) were coated by copoly(DMA-NAS-MAPS) [31] for protein microarrays [32] and by Copoly Azide [17] for the peptide microarray according to the protocol previously described. Briefly, silicon slides were immersed in a polymer solution (1% *w*/*v* in 0.9 M (NH_4_)_2_SO_4_) for 30 min, then rinsed with water, dried under nitrogen and cured for 15 min under vacuum at 80 °C.

Envelope protein E and NS1 antigens are recombinant proteins expressed in *E coli*, >90% pure, purchased from Fitzgerald (North Acton, MA, USA). The proteins were reconstituted to 1 mg/mL in PBS and spotted on copoly(DMA-NAS-MAPS) slides.

Peptides were first dissolved in DMSO to 1uM stock solution and then diluted to the final spotting concentration (50 mM) into the printing buffer for CuACC conjugation on Copoly Azide coated surfaces (25 mM Na/Acetate pH 4.8, 15 mM trehalose, 100 μM CuSO_4_, 400 μM THPTA and 6.25 mM Ascorbic Acid). All the peptide samples were printed in replicates, each sub-array has positional controls (Cy3-streptavidin) and negative controls (printing buffer). Microarrays were arrayed using a non-contact S12 Spotter (Scienion Co., Berlin, Germany). Sixteen arrays were spotted on each silicon slide corresponding to the sixteen compartments created by NEXTERION® IC-16 sixteen well Incubation Chamber from Schott (Jena, Germany). Printed slides were placed in a humid chamber and incubated overnight at room temperature. Then the peptide arrays were immersed in 2 mM EDTA water solution for 1 h whereas protein arrays were blocked by ethanolamine 150 mM in TRIS/HCl 0.1 M pH 9, washed with water and dried under a stream of nitrogen.

Sera were diluted 1:50 in LowCross-Buffer^®^ from Candor (Wangen, Germany) and 40 μL were added into each microarray well, incubated for 30 min on a shaker (150 rpm, 22 °C). Dengue positive and healthy sera were incubated in parallel up to 16 samples per slide. Negative controls included blank arrays incubated only with the secondary antibody and with incubation buffer.

The microarray slide was then rinsed for 3 times with washing buffer (0.05 M Tris/HCl pH 9, 0.25 M NaCl, 0.05% *v*/*v* Tween 20) and PBS and incubated with 40 μL of 1 µg/mL Cy-3 labelled goat anti-human IgG for another 30 min followed by the same washing steps as described above.

For rabbit anti-E01 detection, Cy-3 labelled goat anti-rabbit IgG from Jackson Immunoresearch was used.

Fluorescence was detected by a TECAN Power Scanner. Fluorescence intensities were analysed using the QuantArray software from PerkinElmer and corrected for spot-specific background, values for replicate spots were averaged.

Student *t* tests over the groups of samples were performed using Prism 7 software from GraphPad (Version 7).

### 3.3. Molecular Dynamics Simulations

The X-ray crystal structure of protein E dimer (PDB ID code 4UT6) [33] as used as the starting point for the simulations performed in this study. The protein was solvated in a TIP3P water box whose edges were required to have a minimum distance of 10 Å from the protein surface. A proper number of counter-ions were added to achieve charge neutrality. The system was described by Amber Force Field ff14SB [34] for the protein atoms, the TIP3P model for the water molecules and the parameters proposed by Joung et al. for the counter-ions [35]. To remove initial atom-atom clashes in the protein structure, the system was relaxed using a multi-step protocol: (1) energy minimization for 10,000 steps or until the energy gradient of 0.002 kcal mol^−1^ Å^−1^ was reached and backbone atoms were restrained with a harmonic potential of 20 kcal mol^−1^ Å^−1^; and (2) energy minimization for 20,000 steps or until an energy gradient of 0.002 kcal mol^−1^ Å^−1^ was reached without applying any restraint. Finally, the system was heated to 300 K in 300 ps, and pressure was increased to 1 atm. During MD simulations, temperature and pressure were kept constant using Langevin thermostat [36] and Monte Carlo barostat [37], respectively. The bonds involving hydrogen atoms were constrained by applying the SHAKE [38] algorithm. The cut-off range for non-bonded van der Waals interactions was set to 9.0 Å. Electrostatic interactions were treated using the Particle Mesh Ewald (PME) method [39]. All the calculations were run with the PMEMD code in the GPU accelerated version [40] using a time step of 2 fs. Production trajectories were run for 500 ns in three independent replicas (total 1.5 µs).

The sampled protein conformations were clustered using the gromos method developed by Daura et al. [41], available in the GROMACS package (version 5.0.7) [42]. After several attempts and accurate visual inspection of the outputs, we used an appropriate RMSD cut-off value (2.6 Å) as the optimal threshold to discriminate different conformations populated during the sampling, while at the same time limiting the number of singleton clusters.

### 3.4. Epitope Prediction

Epitopes sequences on protein E were predicted using the Matrix of Local Coupling Energies method (MLCE) [43,44], which combines the analysis of structural/dynamical determinants of a given protein with its energetic properties. This approach allows us to identify non-optimized, low-intensity energetic interaction-networks, corresponding to those substructures that can be more prone to establish interactions with antibodies and suitably recognized by binding partners. The method has been widely described and validated elsewhere [45,46,47,48]. Briefly, the contiguous regions on the protein surface that are deemed to have minimal coupling energies with the rest of the structure are selected on the basis of the eigenvalue decomposition of the matrix reporting the non-bonded interaction of all residue-pairs. The eigenvector associated to the most negative eigenvalue permits to reconstruct a simplified matrix which reports the maximal and minimal stabilizing residue-pairs in the protein structure. Filtering of the simplified matrix with the contact matrix allows us to identify contiguous residue-pairs characterized by their essential degree of coupling to the rest of the protein. The selection of proximal pairs showing minimal coupling with the rest of the protein defines putative epitopes. Selection is carried out on the basis of a threshold value (called softness [43]), which defines the percentage of the set of putative interaction sites by including increasing residue-residue coupling values until the number of couplings that correspond to the lowest contact-filtered pairs under the threshold was reached.

To evaluate the performance of the predictions and relate the physico-chemical properties of epitopes in their native context to observed immunoreactivities of derived peptides, here we performed different experiments 1) by varying the level of prediction softness, 2) by using the domain decomposition energetic matrix [46] and 3) by considering either the monomeric or the dimeric form of E protein. The results from all the experiments were comparatively analysed. Calculations were carried out using the structure of DENV2 E protein deposited in the Protein Data Bank with PDB code 4UT6 [33].

The starting configuration of the protein was refined and minimized by 200 steps of the steepest descend using AMBER 14. The MM-PBSA method (Molecular Mechanics energies combined with the Poisson–Boltzmann and Surface Area continuum solvation) was then applied to obtain the free energy profile stored in the MLCE further exploited to perform epitopes prediction.

### 3.5. Peptide Synthesis

Table 1 reports the sequences of the peptides used in this work.

All peptides were synthesized by stepwise microwave-assisted Fmoc-SPPS (Solid Phase Peptide Synthesis) on a Biotage ALSTRA Initiator+ peptide synthesizer. Briefly, peptides were assembled on a 2-Chlorotrityl chloride (CTC) resin. Chain elongation was performed by iterative cycles of amino acids coupling (using Oxyma/*N*,*N*′-diisopropylcarbodiimide (DIC) as activators) and Fmoc-deprotection using a 20% piperidine solution in *N*,*N*′-dimethylformamide (DMF). Upon complete chain assembly, peptides were cleaved from the resin using a 2.5% thioanisole, 2.5% water, 92.5% trifluoroacetic acid (TFA) mixture. Crude peptides were then purified by preparative RP-HPLC. MS analysis was performed separately on purified material.

## Figures and Tables

**Table 1:** Sequences of peptides predicted and tested for immunoreactivity modified with short-chain polyethylene glycol (PEG) spacers (O_2_Oc) bearing a terminal propargylglicine.

Peptide Code	Peptide Sequence
E01	Prg-(O2Oc)_2_-DRGWGNGCGLFG
E02	Prg-(O2Oc)_2_-TGKFKVVKEIAE
E03	Prg-(O2Oc)_2_-IPFEIMDLEKRHVLGRL

## Supplementary Materials

The following are available online at https://www.mdpi.com/1422-0067/20/8/1921/s1.
