# Supplementary material for: Computational Analysis of Dengue Virus Envelope Protein (E) Reveals an Epitope with Flavivirus Immunodiagnostic Potential in Peptide Microarrays
Source: Int J Mol Sci. 2019 Apr 18;20(8):1921. doi: 10.3390/ijms20081921 (PMC6514720; doi:10.3390/ijms20081921)
Supplement: Supplementary file 1 [file ijms-20-01921-s001.pdf]

Supplementary Material

Computational Analysis of Dengue Virus Envelope Protein (E) Reveals an Epitope with Flavivirus Immunodiagnostic Potential in Peptide Microarrays

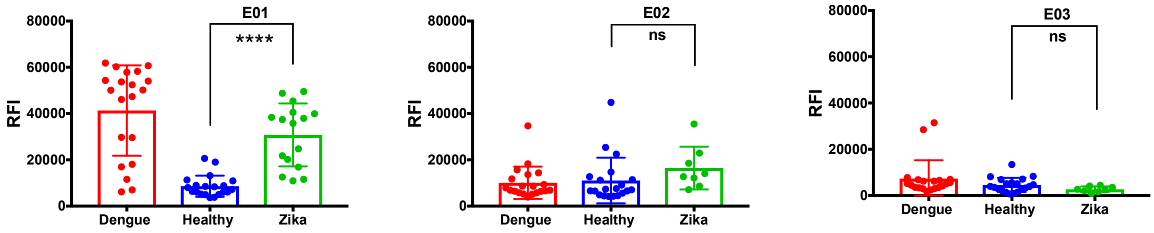

Figure S1. Scatter plots reporting individual and mean immunoreactivity with SD of dengue and zika positive versus healthy control individuals and results of the unpaired *t* test for zika infection diagnosis. ns = not significant. Significant: **** *p* < 0.0001.

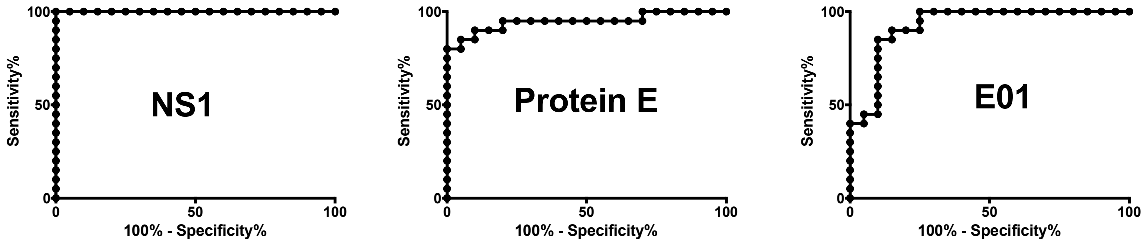

Figure S2. ROC curves for microarray tests of dengue infection using NS1, Protein E and peptide E01.
